# Funding and COVID-19 research in Africa: two years on, are the research needs of Africa being met?

**DOI:** 10.12688/openresafrica.14185.1

**Published:** 2023-09-12

**Authors:** Emilia Antonio, Moses Alobo, Marta Tufet Bayona, Kevin Marsh, Proochista Ariana, Alice Norton

**Affiliations:** 1 Pandemic Sciences Institute, University of Oxford, Oxford, England, UK; 2 Centre for Tropical Medicine and Global Health, University of Oxford, Oxford, UK; 3 Science For Africa Foundation, Nairobi, Nairobi County, Kenya; 4 GAVI Alliance, Geneva, Switzerland

**Keywords:** Africa, COVID-19, Research, Response, Research prioritisation

## Abstract

Background: The Coronavirus disease 2019 (COVID-19) pandemic caused significantly lower reported mortalities on the African continent as compared to other regions. Yet, many countries on the continent are still contending with the devastating economic, social and indirect health impacts. African researchers and policy makers have identified research priority areas which take cognisance of the unique research needs of African countries. A baseline assessment of the alignment of funded research in Africa to these priorities and World Health Organization’s COVID-19 research priorities was undertaken in July, 2020. We present a two-year update to this analysis of funded COVID-19 research in Africa.

Methods: Data captured in the UK Collaborative on Development Research and Global Research Collaboration for Infectious Disease Preparedness COVID-19 Research Project Tracker as of 15th July, 2022 was analysed. An additional analysis of institutions receiving funding for COVID-19 research is presented. We also analysed the change in funding for COVID-19 research in Africa since July, 2020.

Results: The limited COVID-19 research identified in Africa early in the pandemic has persisted over the subsequent two-year period assessed. When number of projects are considered, governmental funders based in Europe and United States supported the most research. Only nine research funders based in Africa were identified. A number of partnerships between African institutions and institutions based on other continents were identified, however, most research projects were undertaken in research institutions based in Africa only. Our findings highlight the relevance of the WHO research priorities for the pandemic response in Africa. Many research questions raised by African researchers remain unaddressed, among which are questions related to clinical management of COVID-19 infections in Africa.

Conclusions: Two years after the identification of Africa’s COVID-19 research priorities, the findings suggest a missed opportunity in new research funding to answer pertinent questions for the pandemic response in Africa.

## Introduction

Since the first case of Coronavirus disease 2019 (COVID-19) was reported in Africa in February 2020, the continent, like the rest of the globe, experienced multiple waves of infections. However, reported direct mortalities from COVID-19 have been much less than projected in Africa at the onset of the pandemic, despite evidence of widespread community transmission ^1^. Noting the variations in estimated deaths across African countries, reported mortalities from COVID-19 were consistently lower in Africa compared to other regions. An estimated 175,315 COVID-19 related deaths had been reported in Africa as of 21 ^st^ March, 2023 representing approximately 2.5% of global mortalities. However, this is likely to be an underestimation due to limited testing and reporting ^2^.

The pandemic and control measures initiated in the pandemic response affected access to health and social care services resulting in significant losses in key gains made in improved child immunisation, maternal and child mortality ^3, 4^. Economic setbacks triggered by the pandemic have contributed to worsening poverty on the continent with associated negative consequences for health and wellbeing. The United Nations estimated an additional 30 million Africans have been pushed into extreme poverty due to the pandemic. Long term sequelae of COVID-19 infections are forecast to burden health systems globally. In Africa, this will put further strain on already fragile health systems weakened by the pandemic and the multiple competing health priorities in the region.

Outstanding successes in research and development (R&D) were witnessed during the pandemic. The first COVID-19 vaccines were licensed within a year of the pandemic ^5^. In April 2020, the Access to COVID-19 Tools (ACT) Accelerator, was initiated to promote global access to COVID-19 diagnostics, treatments and vaccines. The vaccine arm of the ACT Accelerator, COVID-19 Vaccines Global Access (COVAX) is coordinated by Gavi, the Vaccine Alliance, the Coalition for Epidemic Preparedness Innovations (CEPI) and World Health Organization (WHO). COVAX secured billions of COVID-19 vaccine doses to support global vaccination programmes. However, few African countries have not yet attained vaccination levels comparable to coverage in higher income countries in other regions. There has also been disparity in access to novel therapeutics compounding the risk posed by COVID-19 in an under vaccinated population ^6, 7^.

Africa’s strategy for containing the pandemic was coordinated by the Africa Centres for Diseases Prevention and Control which initiated a “ Joint Continental Strategy”. Under this strategy, the Africa Task Force for Novel Coronavirus (AFCOR) was set up involving health ministry representatives of all 55 African Union (AU) member states. The Africa Centres for Disease Control and Prevention (Africa CDC) was also involved in activities to promote COVID-19 R&D in Africa.

### Africa’s COVID-19 research priorities and research priorities for less-resourced countries

In February 2020, WHO and the Global Research Collaboration for Infectious Diseases Preparedness (GloPID-R), a global partnership of funders funding research on disease outbreaks, convened a meeting of experts to identify global research priorities for COVID-19. A *Coordinated Global Research Roadmap: Novel Coronavirus*, outlining these priorities, was published in March 2020. The Roadmap formed the basis for a consultative process led by the Africa Academy of Sciences (AAS) to identify the research areas most pertinent for controlling the pandemic in Africa. The objective of this exercise was to identify regional research needs not captured in WHO’s global framework given the unique contextual considerations for research in Africa. The priorities outlined included investigating management of individuals co-infected with COVID-19 and tuberculosis, developing care protocols for clinical management in the absence of intensive care units (ICUs), and exploring therapeutic options from indigenous herbs or traditional medicines.

The research priority areas identified by the AAS were updated following a collaborative study undertaken by the Global Health Network (TGHN), UK Collaborative on Development Research (UKCDR) and AAS to identify research priorities for Africa and low-resourced settings ^8^. Some of the updated priorities were forward-looking and considered the long-term environmental impact of pandemic control measures, such as lockdowns, and preparedness research for future epidemics and pandemics on the continent.

The research priority lists were reviewed by the Science Standards and Regulatory Technical Working Group of Africa Task Force for Coronavirus (AFTCOR) and summarised into policy actions highlighting areas of greatest research need. The policy paper “ *Research and development priorities for Africa*” was published in February, 2021. These actions were shared with national ministries of health of African Union members, to be factored into national health policies and COVID-19 R&D strategies.

### Tracking funding for COVID-19 research in Africa

In April 2020, UKCDR and GloPID-R launched the COVID-19 Research Project Tracker (the tracker) as part of the COVID-19 Coordination and Learning (COVID CIRCLE) initiative ^9^. The Tracker is a live and regularly updated database capturing new globally funded COVID-R research with projects classified against the WHO research priorities. It is one of the most comprehensive databases which, together with the regularly updated Living Mapping Review (LMR) of projects captured in the Tracker, has been beneficial to researchers, funding organisations and policy makers in identifying gaps in COVID-19 research ^10^. As of 15 ^th^ July, 2022 the database contained data on 17,955 COVID-19 projects funded by 345 funders across 157 countries globally.

In July 2020, we reviewed the Tracker data to assess the scale of funded COVID-19 research involving African countries ^11^. Only 4.5% (of 1858) projects involved at least one African country with the majority of research projects supported by funders based in Europe. The review also assessed alignment of the funded research to the research priorities for Africa and less-resourced countries (as of July, 2020). We undertook a comparative analysis between the Tracker data on one hand, and the WHO International Clinical Trials Registry Platform (ICTRP) and data captured in G-finder on the other. We concluded that there was limited support for research in Africa with limited research on the specific research questions of importance to Africa.

### Study aim

This study is a two-year update to our prior mentioned “baseline” review and provides a more current assessment of funded COVID-19 research in Africa. In addition to previous analyses of funders and alignment of projects to Africa’s research priorities, this update includes an analysis of institutions receiving funding to undertake research in Africa and an assessment of the change in funding over the two-year period. To the best of our knowledge, no similar assessment has been undertaken, and this article offers the most comprehensive analysis of funding for COVID-19 research activities in Africa to date.

## Methods

We reviewed projects captured in the UKCDR and GloPID-R COVID-19 Research Project Tracker. The data fields considered in this work include: project title; abstact; research funder(s); funding amount; research institution(s); and, research location(s). All projects in the Tracker database are also classified to one or more WHO mid- to- long-term COVID-19 research priority areas. Projects which could not be assigned to any of the WHO priority areas were tagged as “N/A” in the database. Further details on the approach to data capture and the remaining data fields in the databsse are published in the LMR protocol in *Wellcome Open Research* ^12^. All projects included in the database as of 15 ^th^ July, 2022 were included in this study ^13^.

The tracker data were downloaded and analysed in *Microsoft Excel (2021)*. The locations of research projects were reviewed to identify projects involving at least one African country (one or more of the 55 African Union member states).

### Funders and research institutions involved in COVID-19 research in Africa

Funders of research involving at least one African country were stratified by location and characterised, following *Google* search and review of funders’ websites, by type of funding organisation (private, public or philanthropic funders). Funding amounts provided for COVID-19 research projects were analysed for each funder identified. Funders involved in co-funded research projects were identified and described. Similarly, research institutions receiving funding to undertake research were grouped by country of location following *Google* search and review of institutional websites.

### Mapping to WHO Research priorities and research priorities of low-resourced countries

Projects involving at least one African country were analysed for alignment to one or more of the nine broad WHO research priority areas and sub-priorities as assigned in the database. Next, by reviewing the research project abstracts and titles, the projects were classified against one or more of the research priorities for Africa and less-resourced countries.

## Results

### Research locations and funders involved in COVID-19 research in Africa

**Figure 1.**
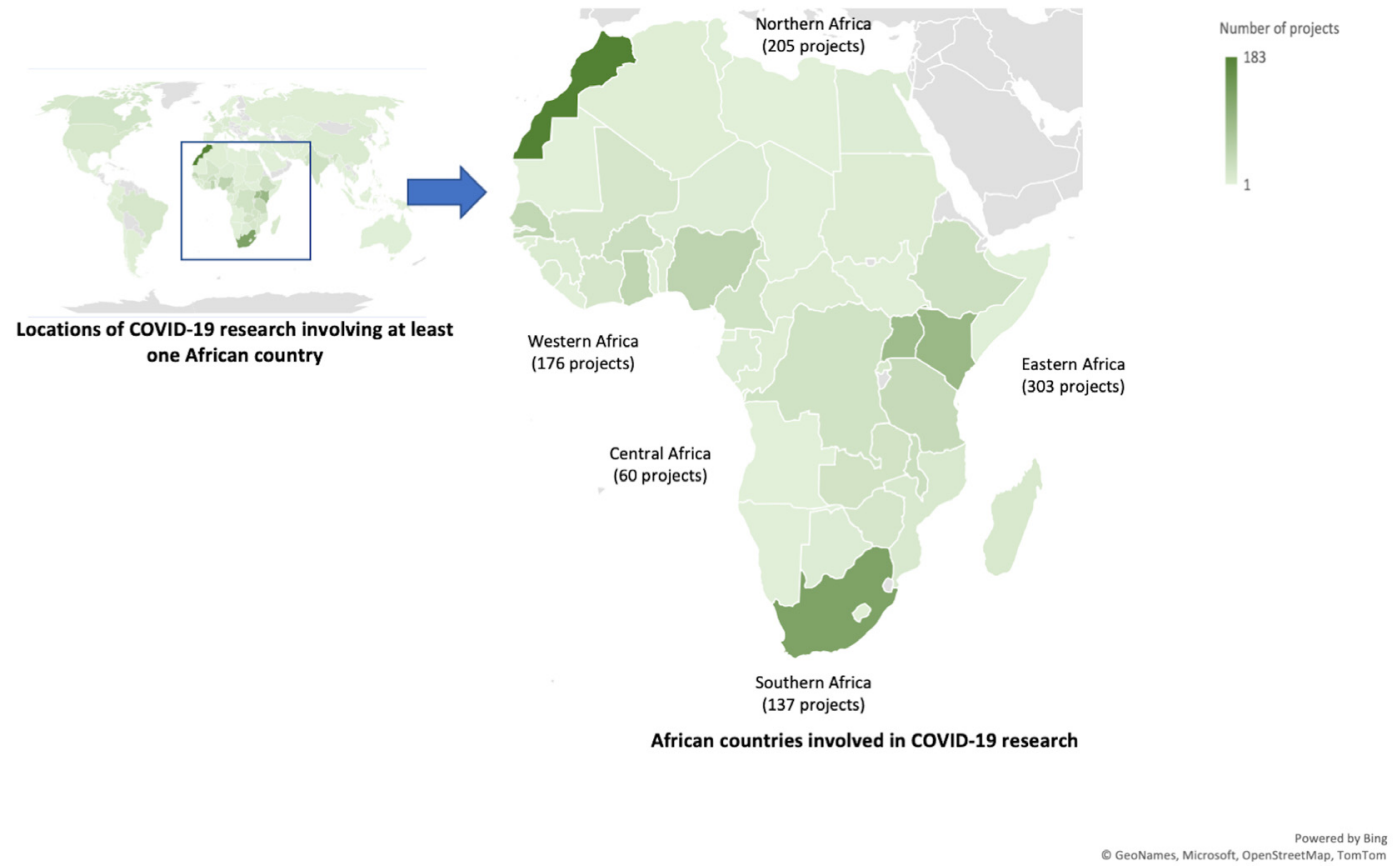
Locations of COVID-19 research projects identified in at least one African country.

Of the 17,995 research projects captured in the UKCDR and GloPID-R COVID-19 Research Project Tracker as of 15 ^th^ July, 2022, only 786 projects were undertaken in at least one African country. Figure 1 shows the 49 African countries involved in COVID-19 research with the majority of projects located in the East Africa Sub-region. The top three countries involved in COVID-19 research projects were Morocco (183 projects), South Africa (128 projects), and Kenya (91 projects).

**Figure 2.**
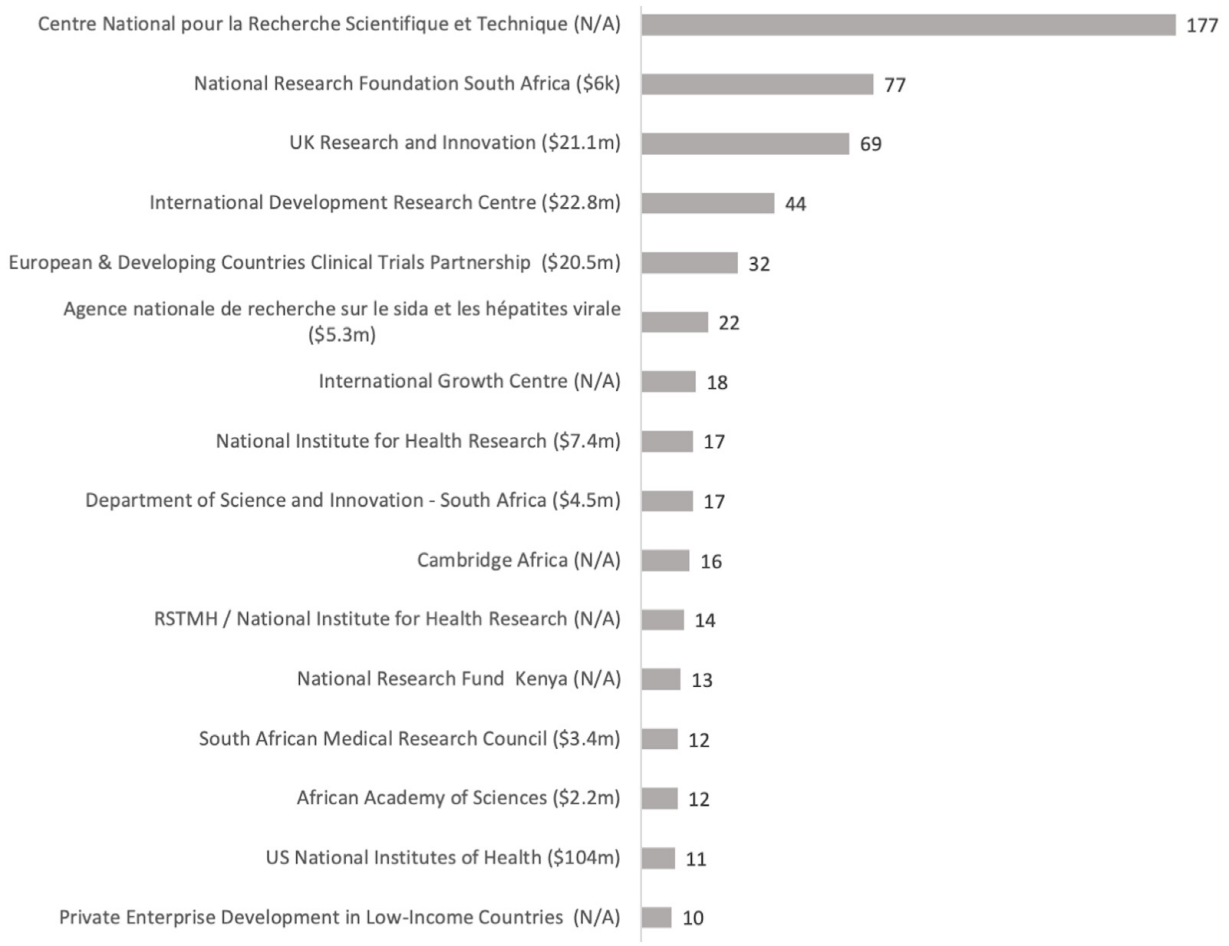
Funders of COVID-19 research in Africa with known funding amounts and number of projects shown. **Known funding amounts in brackets, funders of 10 or more projects shown. 39% of research projects in Africa include details on funding amounts. N/A is indicated where no information on funding amounts identified. **Funders of 10 or less projects**: IHU **Marseille** (N/A, 1 project); **York University** ($10.6k, 1 project); **IRD** (N/A, 1 project); ANRS / Expertise France ($132k, 1 project), IZA – Institute of Labor Economics (N/A, 1 project), AXA (N/A, 1 project); Johns Hopkins University (N/A, 1 project); **CHEST Foundation** (N/A, 1 project); **KAKENHI** ($143k, 1 project); **DFG** (N/A, 1 project); **Mitacs** (N/A, 1 project); European Commission ($2.9m, 1 project); **National Science Center Poland** ($80.4k, 1 project); **ICMR / National Institute for Health Research** **/ UKRI** ($240k, 1 project); **NSF** (USA) ($169k, 1 project); **AFD / IRD** (N/A, 1 project); **SNSF** ($53k, 1 project); **BSAC** ($22.7k, 1 project); **Solidarity Fund/Michael and Susan Dell Foundation** ($910k, 1 project); **EOSC** (N/A, 1 project); **Spencer Foundation** (N/A, 1 project); **ICMR / NIH** ($3.7k, 1 project); **SSHRC** ($712k, 1 project); **CIRAD** (N/A, 1 project); **UNITAID / ANRS** (N/A, 1 project); **ANRS-MIE** (N/A, 1 project); **UNITAID / EDCTP** (N/A, 1 project); **FCDO (formerly DFID) / UNICEF** (N/A, 1 project); **WHO** (N/A, 1 project); **International Science Council** (N/A, 2 projects); **Other Funders (Canada)** (N/A, 2 projects); **RSTMH** ($13.4k, 2 projects); **Solidarity Fund** ($142k, 2 projects); **ICGEB** (N/A, 2 projects); **Burnet Institute** (N/A, 2 projects); **European Commission (Horizon)** ($35.8m, 2 projects); **Innovations for Poverty Action** (N/A, 2 projects); **Newton Fund** ($151k, 2 projects); **Swedish Research Council** ($977m, 2 projects); **South African Medical Research Council/Department of Science and Innovation – South Africa** ($1.1m, 3 projects); **WHO / Gabon government** (N/A, 3 projects); **Volkswagen Stiftung** (N/A, 3 projects); **BMBF** ($753k, 4 projects); **Agence nationale de la recherche (ANR)** (N/A, 4 projects); **Wellcome / FCDO (formerly DFID)** ($5.6m, 4 projects); **COVID-19 Therapeutics Accelerator (Wellcome / Bill & Melinda Gates Foundation)** ($N/A, 4 projects); **WHO Africa** ($N/A, 4 projects); **British Academy** ($25k, 4 projects); **Duke University** (N/A, 4 projects **); Partnership for Economic Policy** (N/A, 5 projects); **Innovations for Poverty Action/ FCDO (formerly DFID)** (N/A, 5 projects); **MRIC Mauritius** ($74k, 6 projects); **CIDRI-Africa** ($338k, 6 projects); **RCN Norway** ($2.3m, 6 projects); **Wellcome** ($1.4m, 6 projects); **G2LM|LIC** (N/A, 7 projects); **BRICS** (N/A, 7 projects); **UNICEF** (N/A); **AUF** (N/A, 7 projects); **FCDO (formerly DFID) / National Institute for Health Research / Wellcome (Elrha funding call)** ($988k, 8 projects); **Novo Nordisk Foundation** (800k, 8 projects); **AFD** ($12.6m, 8 projects); **RAENG** ($210k, 8 projects); **Social Sciences Research Council** (N/A, 9 projects); **UKRI / National Institute for Health Research** ($2.3m, 9 projects); **Department of Science and Innovation – South Africa /Technology Innovation Agency** ($1.6m, 9 projects); **CIHR** ($3.3m, 9 projects); **Institut Pasteur** (N/A, 9 projects).

At least USD 267million was invested in COVID-19 research in Africa by 75 research funders based in 17 countries. The majority the funders are public or governmental funding organisations in Europe and the USA with National Center for Scientific and Technical Research, National Research Foundation South Africa and UK Research and Innovation funding the most research ( Figure 2). A total of nine funders based in Africa were identified ( Table 1). Most projects were funded by one funding organisation. Of the 63 co-funded projects, UK NIHR was involved in the most co-funding partnerships, followed by Royal Society of Tropical Medicine, UK and the Foreign Commonwealth and Development Office, UK.

**Table 1.** Funders of COVID-19 research in Africa based on the African continent.

Funding organisation/ funder	Country of organisation	Type of funder
African Academy of Sciences	Kenya	Not for profit
CNRST	Morocco	Public
Technology Innovation Agency	South Africa	Public
MRIC Mauritius	Mauritius	Public
NRF Kenya	Kenya	Public
NRF South Africa	South Africa	Public
South African Medical Research Council	South Africa	Public
Department of Science and Innovation	South Africa	Public
Gabon government	Gabon	Public

### Institutions involved in COVID-19 research in Africa

Details on institutions leading research were available for 570 (72%) of the research projects in Africa. Of these, 179 (31.4%) were led from only institutions based outside Africa. The remainder of the projects involved only African institutions (62.8%) or involved both institutions in Africa and those located outside the continent (5.8%). Most projects had a single lead institution listed, and for 41 projects multiple (more than one) lead institutions were identified.

**Figure 3.**
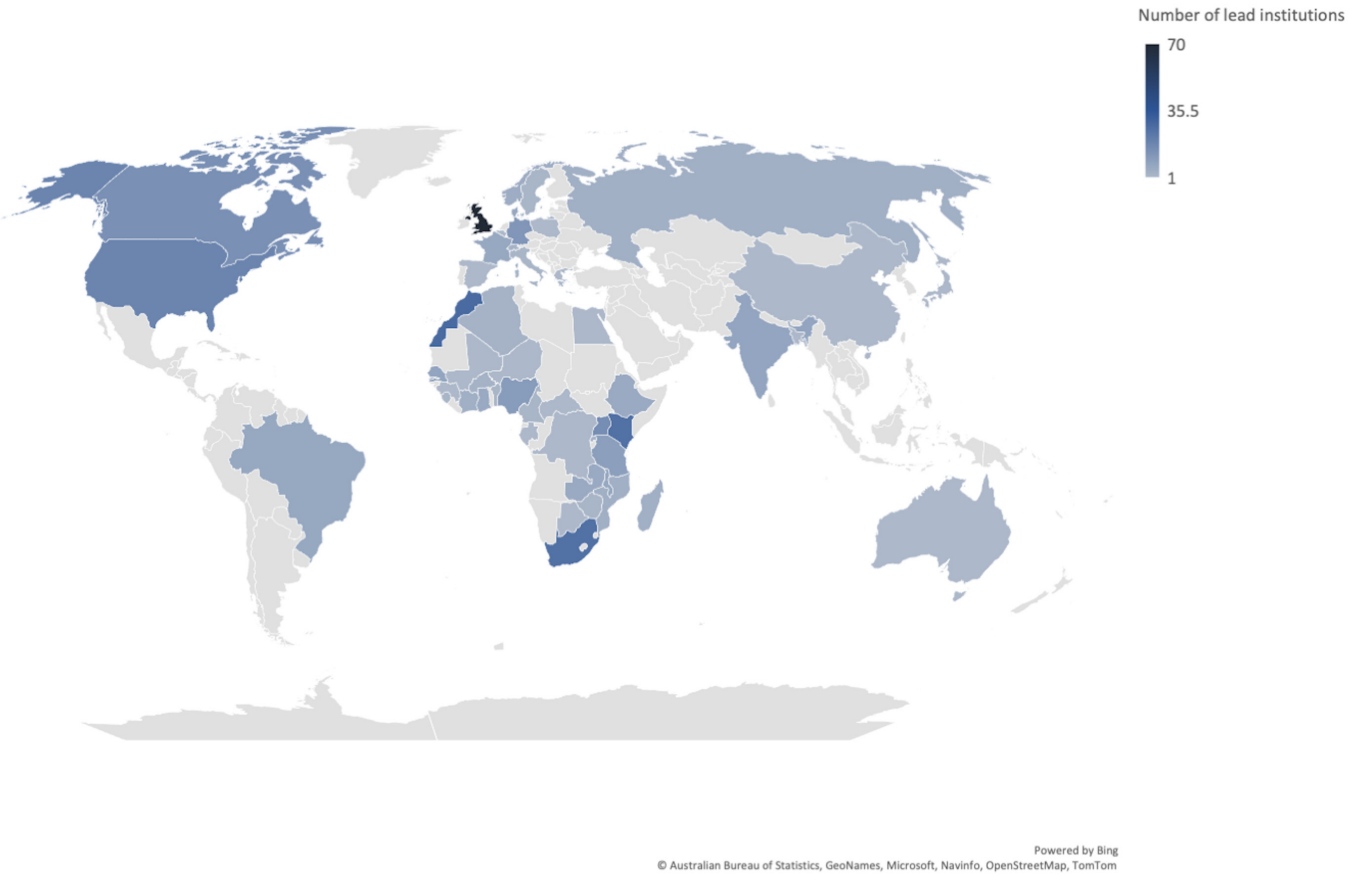
Countries of institutions receiving funding for COVID-19 research projects in Africa. **72% of projects include information on lead institution.

**Figure 4.**
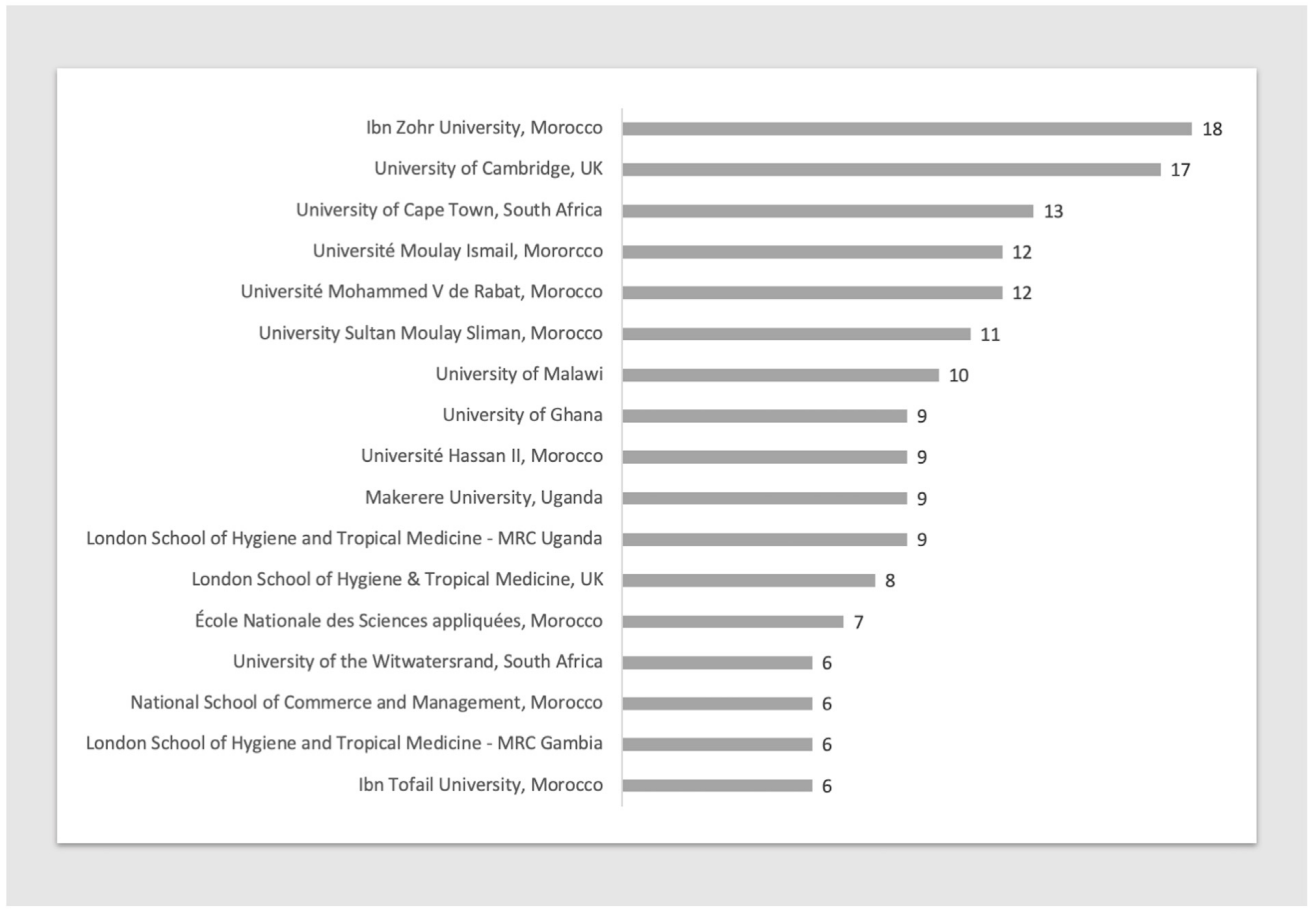
Top 10 institutions (based on number of projects) receiving funding for COVID-19 research in Africa.

A total of 365 institutions in 54 countries received funding for COVID-19 research projects involving at least one African country ( Figure 3). Most of these are in the United Kingdom (70 institutions), Morocco (29 institutions), Kenya and South Africa (26 institutions each). The Ibn Zohr University (Morocco) was listed as lead institution for the highest number of research projects taking place in Africa. This was followed by University of Cambridge (UK) and University of Cape Town, South Africa ( Figure 4).

## Mapping to WHO research priorities and research priorities for less-resourced countries

### WHO research priorities

**Figure 5.**
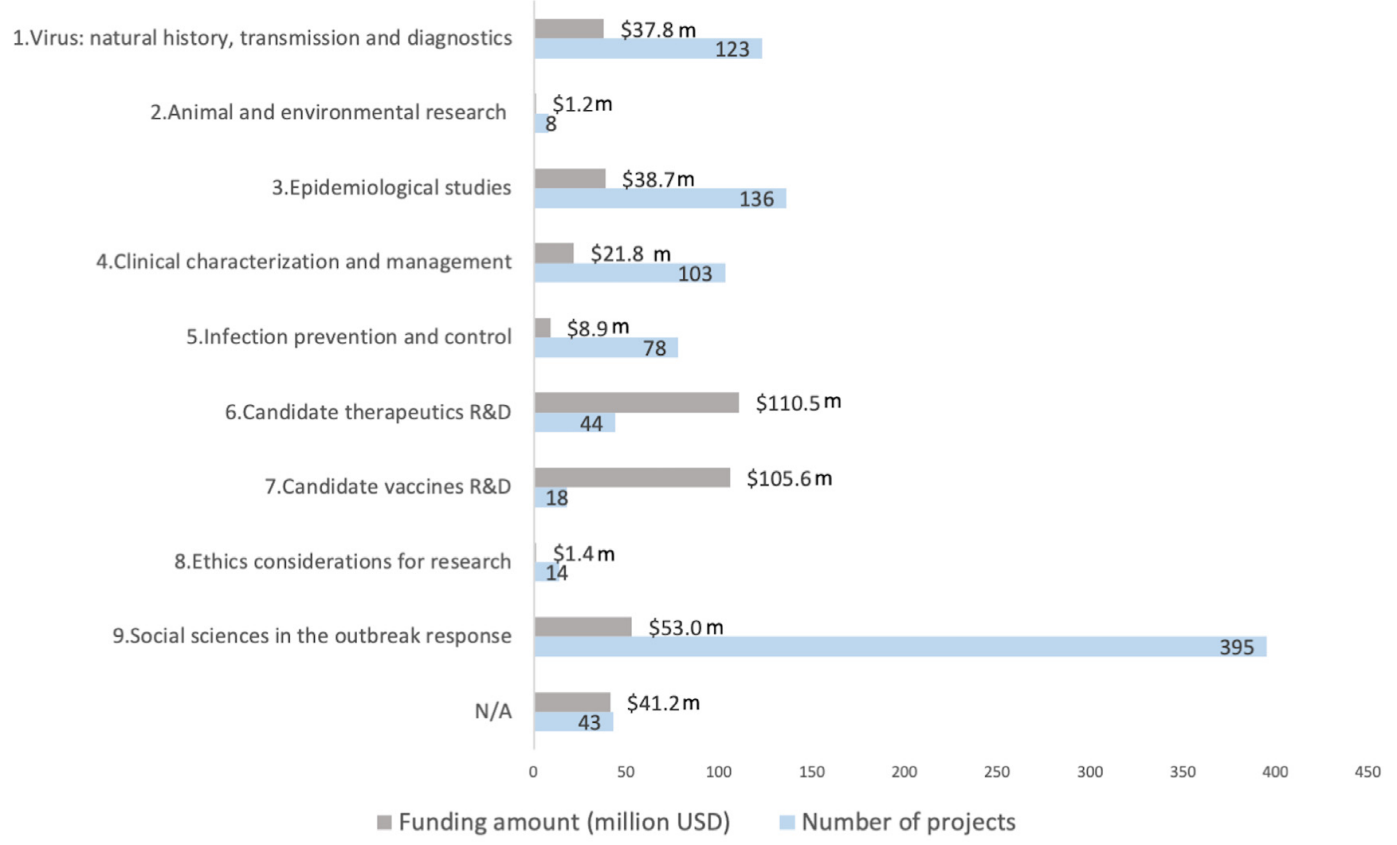
COVID-19 research projects in Africa classified against the nine broad WHO COVID-19 research priority areas. **Some projects map to multiple priority areas. Known funding amount invested in each priority area shown.

**Figure 6.**
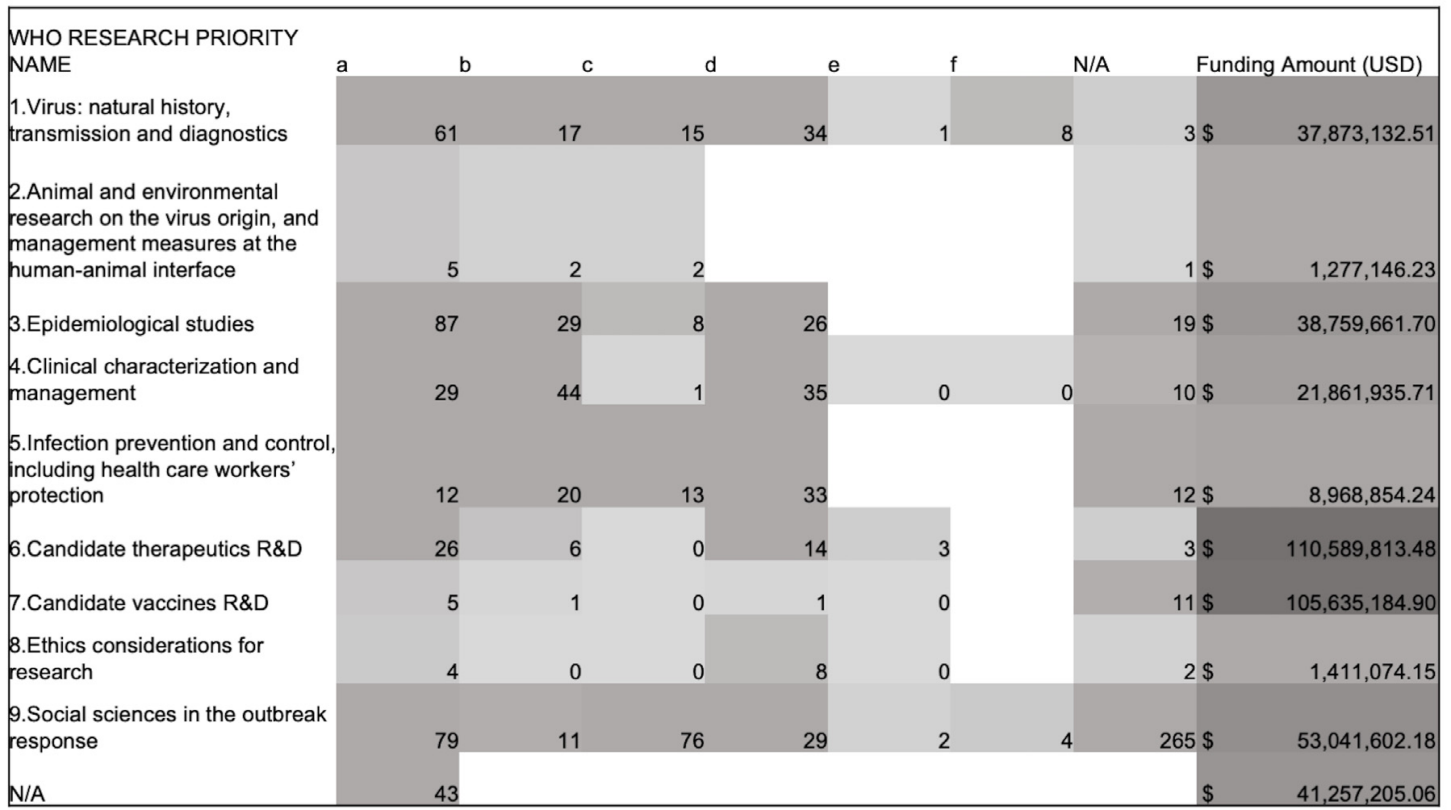
COVID-19 research projects in Africa classified against the WHO COVID-19 research priorities and sub-priority areas. **Some projects map to multiple priority areas. Known funding amounts invested in each priority area shown.

Based on the primary area of research focus, projects were classified against the WHO research priority areas as shown in Figure 5. In addition, projects were classified to the corresponding sub-priorities in Figure 6. In total, 95% of the projects mapped to at least one of the nine broad WHO research priority areas. “Social sciences in the outbreak response”, “Epidemiological studies” and “Virus: natural history, transmission and diagnostics” were the top three research priority areas which most projects mapped to.

The majority of the projects under “social sciences in the outbreak response” did not map to any of the social science sub-priority areas and were designated “N/A”. For the projects which could be classified, most were on measures to promote uptake of public health interventions and effective modes for communication during the pandemic. Under “epidemiological studies” understanding coronavirus transmission dynamics was the dominant area followed by identifying individuals at high risk of severe COVID-19 infections and research into the role of children in COVID-19 transmission.

Research for the development of diagnostics, understanding immunity to COVID-19 and understanding the disease pathogenesis were the top three areas of focus of research projects under “virus: natural history, transmission and diagnostics”. The highest funding amounts invested were for vaccines and therapeutics R&D. However, only a few projects mapped to these two priority areas. “Ethics considerations for research” and “animal and environmental health” were the two priority areas with the least number of research projects.

### Research priorities for Africa and less-resourced countries

**Figure 7.**
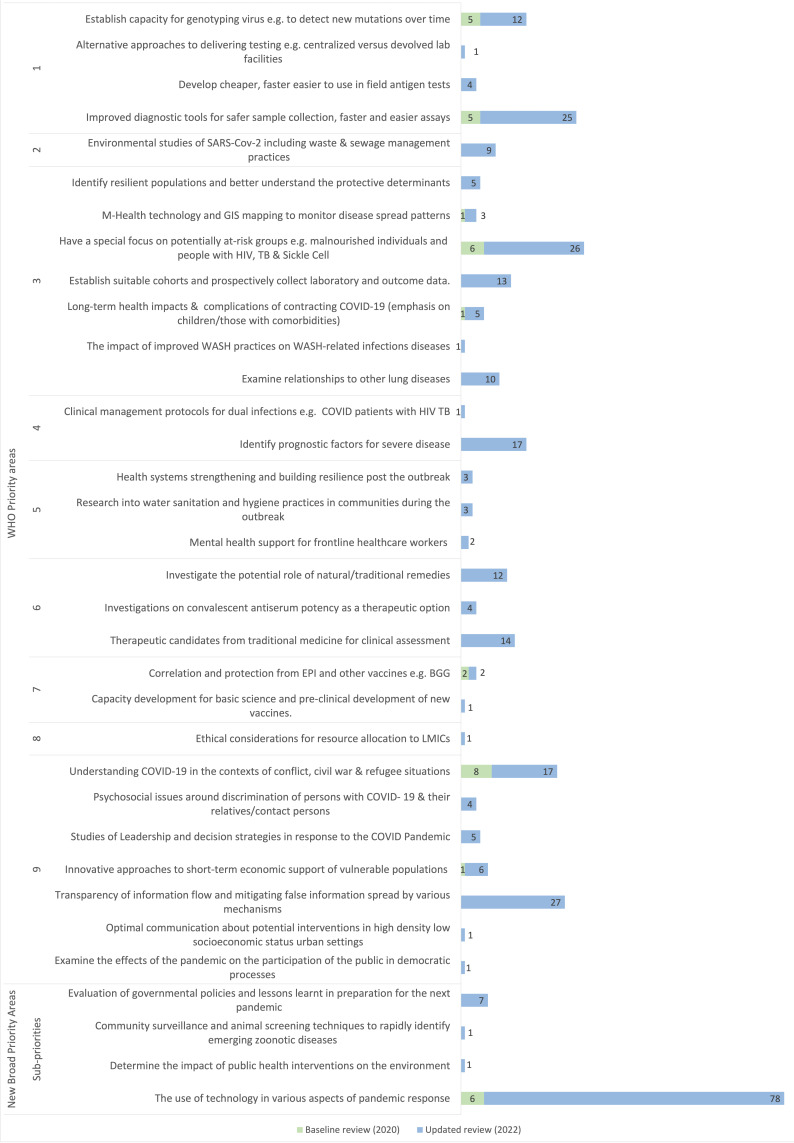
COVID-19 research projects in Africa mapped to the COVID-19 research priorities of Africa and less-resourced countries. **Some projects map to multiple priority areas. Results from baseline mapping done in 2020 and this update (2022) shown.

Few projects focussed on the research priorities of Africa and less-resourced countries as shown in Figure 7. The dominant area of research was on the use of technology in the pandemic response. The next three areas of focus of most projects were: investigating methods of transparent flow of information and mitigating misinformation; research in at-risk populations such as the malnourished, persons living with HIV, Tuberculosis (TB) and Sickle cell disease; and, improved COVID-19 diagnostic tools. Of the research priorities for which no projects were identified, most were on clinical management of COVID-19 including development of clinical management protocols in contexts with limited or no ICU capacity, community rehabilitation modalities post-hospitalisation with COVID-19 and the impact of COVID-19 on the management of other infectious diseases.

### Change in funding over time

Key changes in funded research in Africa over the two-year period are summarised in Table 2. An additional 702 research projects involving African countries were identified in this analysis. Projects involving 11 additional countries than in our prior work were also identified. The known funding amounts invested increased from USD 22 million to 267 million as did the funders investing in research in Africa which increased from 12 to 75 funders.

**Table 2.** Change in funding landscape of COVID-19 research in Africa from July 2020 to July 2022.

	Baseline analysis (2020)	Current study (2022)
UKCDR & GloPID-R Tracker version	July, 2020	July, 2022
Number of projects taking place in African Countries	84 of 1858 total projects (4.5%)	786 of 17,955 (4%)
Number of African countries	38	49
Known funding amounts	USD 22million of 726 million global investment (3%)	USD 267million of 6.5 billion global investment (4%)
Number of funders	12	75
Number of funders based in Africa	None based in Africa	9

UKCDR: UK Collaborative on Development Research, GloPID-R: Global Research Collaboration for Infectious Diseases Preparedness

## Discussion

### Research locations and funders involved in COVID-19 research in Africa

As a proportion of total projects funded globally, few COVID-19 research projects took place in African countries. This is similar to findings of the baseline review undertaken in 2020. Morocco, South Africa and Kenya were the countries involved in the most COVID-19 research projects on the continent and were also among the leading countries identified in bibliometric assessments for COVID-19 research outputs ^14, 15^.

Data from 11 more countries than in the prior assessment are included in this study. However, no research activities involving Burundi, Djibouti, Eritrea, Eswatini, Sahrawi Republic, São and Tomé and Príncipe were identified. Data on research activities in these countries might not have been made publicly available resulting in a lack of capture in the database analysed. The persistent challenges associated with obtaining data on health-related activities in Africa are well recognised. Among the multiple factors contributing to this are poor culture of recording data and limited investment in data systems ^16^.

Most COVID-19 research on the African continent identified was supported by funders based outside the continent in Europe and the USA. This preponderance for health research funding investments to originate from sources external to the African continent poses a potential challenge for ensuring appropriate research agenda are addressed as local research needs may differ from external agenda.

Only nine research funders based in Africa were identified in this analysis, an increase from the 2020 review where none were identified. These funders are based in five African countries including Moroco, Kenya and South Africa, the three countries where most COVID-19 research projects were located. All but one of the nine funders is a governmental funder. These findings, representing mostly new funding awarded for COVID-19 research, support the perception of limited investments by African country governments for health R&D on the continent ^17^. African countries invested only 0.5% of GDP in R&D as of 2018, falling short of the 1% target proposed at the 2007 Africa Union summit. A WHO review of public financing of health in Africa in 2016 suggested there is no association between countries’ wealth and prioritisation of investments in health. Thus, the onus is on African country governments to be more strategic in the allocation of resources for health and health R&D.

The potential benefits of some co-funding models include reducing the cost of administering grants and minimising duplication of research. Only 8% of COVID-19 research projects in Africa were co-funded by two or more funding organisations. This could indicate a potential missed opportunity for greater efficiency of funding allocated for research in Africa.

### Institutions involved in COVID-19 research in Africa

Lead institutions on research grants are typically those institutions which are responsible for grants and, hence, the delivery of the activities for which the grants were awarded. In research partnerships, lead institutions may play a variety of roles including administering funds to partner institutions, coordinating research activities among partners, and leading on grant reporting. As the information captured in the Tracker database lacks sufficient granularity for clarifying roles of institutions named, this analysis reports on all institutions receiving funds to undertake COVID-19 research in Africa.

University of Cambridge and London School of Hygiene and Tropical Medicine were among the top 10 institutions in receipt of funding for COVID-19 research in Africa. All but one of the projects involving the University of Cambridge is funded by the Cambridge Africa programme, which supports long-term partnerships between the University of Cambridge and African institutions. Other instances of similar North-South partnerships were seen for European and Developing Countries Clinical Trials Partnerships (EDCTP) funded research projects and projects funded by the UK MRC supported activities in the Medical Research Council (MRC)/UVRI and London School of Hygiene & Tropical Medicine (LSHTM) Research Units in Uganda and the Gambia. These demonstrate the importance of building and sustaining long-standing relationships among research institutions which can be harnessed in response to outbreaks.

For the COVID-19 research projects in Africa with only institutions based outside the continent identified to have received funding, it is highly likely that local collaborators for the conduct of research were not listed. As funders play an essential role in setting the tone for partnership relationships, it is important that due recognition be given to all research collaborators particularly in the provision of data on funding activities. 

## Mapping to WHO research priorities and research priorities for Africa and less-resourced countries

### WHO research priorities

Almost all the COVID-19 projects identified in Africa mapped to at least one of the broad WHO priority areas. This suggests the WHO research priorities were relevant for the control of the pandemic in Africa, as was similarly concluded following the activities to identify research priorities for Africa in 2020.

The dominant area, “social sciences in the outbreak response”, included many projects which could not be classified under any of the six sub-priority areas. Some of these projects focussed on economic impacts of the pandemic, health systems research and other longer term research questions which were likely not considered to be priorities early in the pandemic when the WHO Roadmap was developed. The questions addressed by many of these projects are covered in the UN Research Roadmap for the COVID-19 Recovery. Published in 2021, it takes a more forward-looking perspective and outlines research areas important for the recovery from COVID-19.

Research on “epidemiological studies” and “virus: natural history, transmission and diagnostics” were the next two areas which most projects focussed on. In the aftermath of the devastating 2014–2016 West Africa Ebola outbreak, many African countries benefitted from initiatives to strengthen laboratory and surveillance capacity ^18^. These efforts, and capacity developed rapidly at the onset of the pandemic, played a vital role in Africa’s response, enabling African scientists to contribute to global genomics databases and enhancing surveillance capacity to detect novel variants ^19^. An example is the Omicron variant of COVID-19 first detected in South Africa ^20^.

When the entire database of funded projects is considered, the least number of projects fell under “Ethics considerations for research” and “animal and environmental research”, which have also received less funding than the other research priority areas ^10^. Under “Ethics considerations for research” the WHO Roadmap includes activities already implemented by WHO itself such as development of guidance for ethical conduct for research. Further, the sub-priorities under “Ethics considerations for research” include non-research actions which could account for the low number of research projects in this priority area. “Animal and environmental research” is a recognised gap in funded research globally ^10^. This analysis suggests a greater focus is also required in this area in Africa.

As COVID-19 was a novel pathogen, there was great urgency to identify effective countermeasures to infections when the outbreak began. Developing effective vaccines and therapeutics were a high priority area for research. It is likely that funding for vaccines and therapeutics R&D was concentrated in better-resourced institutions, typically located in higher income countries. These were equipped to rapidly generate high-quality evidence and could better compete for funding for R&D resulting in significantly fewer vaccines and therapeutics R&D projects in Africa ^21^. A number of vaccines and therapeutics have been licenced for use globally. It is important that African countries are included in research for assessing effectiveness of these products as the control of COVID-19 in Africa has implications for the global control of infections.

### Research priorities for Africa and less-resourced countries

Given that activities to define the priorities for less-resourced countries drew on initial efforts to determine the research priorities for Africa in 2020, this work maps the COVID-19 research projects involving Africa to the research priorities of less-resourced countries. This list of priorities encompasses all the research priorities for Africa identified in 2020 and was the basis for the prior baseline assessment.

Only a few funded COVID-19 research projects focussed on the specific research priorities for Africa and less-resourced countries two years after the identification of research priorities for Africa. The top areas of research focus identified in the 2020 review remain dominant and include developing diagnostics for COVID-19 detection, a focus on persons living with HIV, TB and Sickle cell and, understanding infections in conflict-affected settings. Some new areas previously not represented, such as identifying factors associated with severe disease and identifying therapeutic candidates from herbal and traditional remedies, were found to be the focus of some projects in this review.

Unsurprisingly, many projects in Africa sought to identify effective modes of communication to curtail false information on various aspects of the pandemic, which was a major issue globally and in Africa. Historically, poor communication and community engagement have negatively impacted on outbreak response measures on the continent ^22^. The findings in this review might indicate the recognition of value of research in this area in effectively responding to the pandemic.

For a number of research priority areas of relevance to Africa, no research projects were identified. Research was lacking on clinical research areas such as: development of clinical care protocols for management of severe infections (particularly in the absence of intensive care facilities); community rehabilitation following recovery from COVID-19; and, assessing the impact of COVID-19 on the management of other infectious diseases. These and the areas with limited research focus represent potential research gaps on COVID-19 in Africa. There is a risk that some of these questions might not be answered owing to the finite yet unpredictable window in which certain types of research can be conducted during an outbreak.

## Change in funding over time

This paper shows the evolution of funding for COVID-19 research in Africa since 2020. The limited number of research projects involving African countries as of July, 2020 could have been indicative of a slow response to pandemic in Africa. This could be attributed to the direction of resources to the other regions where the burden of COVID-19 infections was apparently higher. As the pandemic progressed with increased funding calls for COVID-19 research in Africa and low and middle income countries, it is unsurprising to find a significant increase in funded research two-years following our initial work. There was a more than ten-fold increase in funding invested when the known funding amounts were analysed. Several more funders supported COVID-19 research as of July, 2022 than in July, 2020.

## Limitations

The limitations to the data and approaches to data capture for UKCDR and GloPID-R COVID-19 research project Tracker are outlined in *Wellcome Open Research* and in the prior analysis of funded COVID-19 research projects in Africa ^12^. They include insufficient information on research projects, which impacts the manual assignment of some research fields in the database. Another is the sourcing of data directly from funders or from publicly available sources, which has implications for the scope of projects captured. Hence, this analysis is similarly limited by the degree of completeness of data captured in the Tracker database. Where required, we have provided the proportion of projects with data available in the database in the figures presented in this work.

Further, the Tracker is limited in capturing: repurposed research grants (grants awarded pre-pandemic which were pivoted for the COVID-19 response); wider funding for supporting the research system; and, research grant awards which were not publicly available or not provided by the research funders. Therefore, this analysis is likely to better represent an assessment of “new” funding awarded for the COVID-19 research response, particularly data in the public domain.

As we transition into recovery mode, it is likely that many COVID-19 specific research funding calls might have been incorporated into funders’ regular funding portfolios. This poses a challenge to identification of these projects for capture in the database and, hence, could impact on the scope of funded research identified from these analyses.

## Conclusion

This two-year update to the assessment of funded COVID-19 research projects in Africa showed significantly more COVID-19 research activity on the African continent. The pandemic evolved through multiple phases and has been determined to no longer constitute Public Health Emergency of International Concern. There remain pertinent context-specific research questions to be addressed in Africa. For instance, evidence is still lacking regarding which factor or combination of factors accounted for the milder trends in mortality witnessed in Africa. There is a potential risk of a missed opportunity for generating evidence on the continent given the finite window for undertaking research during an outbreak.

## Data Availability

Harvard Dataverse: A living mapping review for COVID-19 funded research projects: two year update. https://doi.org/10.7910/DVN/95IWXT ^13^. Data are available under the terms of the Creative Commons Zero "No rights reserved" data waiver (CC0 1.0 Public domain dedication).
