# Supplementary material for: Funding and COVID-19 research in Africa: two years on, are the research needs of Africa being met?
Source: Open Res Afr. 2023 Sep 12;6:16. [Version 1] doi: 10.12688/openresafrica.14185.1 (PMC10762293; doi:10.12688/openresafrica.14185.1)
Supplement: Peer review history

# Reviewer report: Reviewer response for version 1

**DOI:** 10.21956/openresafrica.15342.r30386

**Published:** 2024-01-02

**Authors:** Carter Bloch

**Affiliations:** 1 Aarhus University, Aarhus, Denmark

This paper maps the funding of COVID-19 research in Africa up until July 2022. The paper is an update of an earlier paper by the authors, where they covered funding in the period up to July 2020. The paper is structured along similar lines to their earlier paper and the authors reach the same conclusion in this current paper, that only a small share of funded COVID-19 research projects involve African research institutions. The share is actually unchanged from the earlier period, at around 4.4% of total COVID-19 research projects.

 The paper provides important documentation of trends in funding of COVID-19 research, however while the paper makes the important point that only a small share of funding includes African researchers, it would be interesting to delve more deeply into the broadening of COVID-19 research funding in Africa. I provide some examples below.

 The introduction could be better linked to the rest of the paper. Currently, it doesn’t really lead into the paper’s objective and what it covers.

 A question posed in the paper is whether Africa’s COVID-19 research needs are being met, however the paper could be more clear on what it finds. For example, it is not fully clear to me which research areas are priorities for Africa and other less-resourced countries. Which of the areas listed in figure 7 are prioritized areas? All of them? On page 6 it is stated that few projects focused on these priorities, but I can’t see what this is based on in figure 7. All in all, greater discussion of this would be helpful. In addition, if possible, it would be good to calculate the total numbers/shares of research within these priority areas (even if they do not involve African research institutions).

 The paper rightly points out that only a small number of funders are located in Africa, however I think it is also worth noting that (according to figure 2) many of these African funders are among the largest in terms of number of funded projects.

 I am not sure I understand figure 6. For example, what do the columns a, b, c, d, etc refer to?

Is the work clearly and accurately presented and does it cite the current literature?

Yes

If applicable, is the statistical analysis and its interpretation appropriate?

Yes

Are all the source data underlying the results available to ensure full reproducibility?

Yes

Is the study design appropriate and is the work technically sound?

Yes

Are the conclusions drawn adequately supported by the results?

Yes

Are sufficient details of methods and analysis provided to allow replication by others?

Yes

Reviewer Expertise:

Research and innovation analysis and policy

I confirm that I have read this submission and believe that I have an appropriate level of expertise to confirm that it is of an acceptable scientific standard.

# Reviewer report: Reviewer response for version 1

**DOI:** 10.21956/openresafrica.15342.r30441

**Published:** 2023-12-28

**Authors:** Chen Dun

**Affiliations:** 1 Johns Hopkins University, Baltimore, Maryland, USA

This is a well-written article described COVID-19 related research project in Africa and drew a conclusion of that there is a missed opportunity of COVID-19 research there which could benefit the future decision and policy making.

 There are a couple thoughts that might be consider: 

 1. What about other research project that is on-going that in Africa? Due to the pandemic, a lot of research project made changes during that time, and how can you also add these weights in?

 2. What are the funding amount? Maybe need to re-think about the comparing measurement? e.g. some countries had more population that had a high number of projects but less funding amount?

 3. Maybe another comparison between African overall vs other countries, and also compare before and after covid period would be beneficial.

Is the work clearly and accurately presented and does it cite the current literature?

Yes

If applicable, is the statistical analysis and its interpretation appropriate?

Yes

Are all the source data underlying the results available to ensure full reproducibility?

Yes

Is the study design appropriate and is the work technically sound?

Yes

Are the conclusions drawn adequately supported by the results?

Yes

Are sufficient details of methods and analysis provided to allow replication by others?

Yes

Reviewer Expertise:

Public health

I confirm that I have read this submission and believe that I have an appropriate level of expertise to confirm that it is of an acceptable scientific standard.
